# Collectanea

**Published:** 1833-05

**Authors:** 


### COLLECTANEA.

**Floriferis ut apes in saltibus omnia libant,**
**Omnia nos, itidem, depascimur aurea dicta.**

### PHYSIOLOGY.

### *On the Umbilical Vesicle.* By M. Mayer.

The results from the observations of this author are, 1st, that, in the normal state, the umbilical vesicle of the human embryo remains visible from the commencement until the end of the entire development of this last. In the placenta of two full-grown twins, two vesicles are easily to be perceived. 2d. That the conduit of the umbilical vesicle becomes permeable only three or four weeks after the descent of the ovum into the uterus. 3d. That the vesicle contains no yellowish, powder-like substance; its conduit, besides being quite whole and permeable, is so narrow that it could serve but very little to the nutrition of the embryo, though the latter is of very small dimensions in the four first weeks of its development. Also it should be remarked, that the umbilical vesicle is large in the carnivora, while it is very small, and could scarcely contain any drops of liquid in the herbivora; in man it is small. 4th. Numerous observations lead us to conclude that the circulation of the blood continues for a long time in the omphalo-mesenteric vesicles; whilst the permeability of the conduit of the umbilical vesicle exists only until the third month of gestation. In a monstrous fœtus of the full time, the omphalo-mesenteric vesicles were very distinct in all their course.—[*Allgem. Medicinische Zeitung.*]

### PATHOLOGY.

### *Pathology of the Nervous System.* [From a Lecture of Professor Graves, Dublin.]—*London Med. and Surg. Journal.*

Some circumstances have, of late, drawn my attention to the study of nervous pathology; and, as this is an interesting subject, and one on which my opinions differ from some of those generally received, a few remarks on this point may not, perhaps, be unacceptable. The observations I am about to make will involve the consideration of the general principles suited to guide us in the difficult study of nervous affections, rather than the description of any particular disease. In considering the symptoms that accompany diseases of the nerves, pathologists have directed their attention almost exclusively to the nervous centres, and have looked on the brain, cerebellum, and spinal cord as the parts in which the causes of all nervous disorders reside, or in which they originate.

If you examine the works of Rostan, Lallemand, Abercrombie, and all those who have written on diseases of the nervous system, you will find that their inquiries consist in searching after the causes of functional changes, either in the cerebrum, cerebellum, or spinal marrow, forgetting that these causes may be also resident in the nervous cords themselves, or their extremities, which I shall call their *circumferential parts.* When we recollect the manner in which the nervous system grows, when we call to mind the fact, that in the development of that system, during the fœtal state, the nervous extremities and trunks are formed before any traces of the brain are discernible, we must at once allow it is by no means improbable that these parts may become incapable of discharging their functions in consequence of changes originating in themselves, and not proceeding from the nervous centres. In a word, may not the decay and withering of the nervous tree commence occasionally in its extreme branches? and may not a blighting influence affect the latter, while the main trunk remains sound and unharmed? In fact, gentlemen, pathologists have, with respect to diseases of the nervous system, committed an error precisely similar to that which was so long prevalent with regard to diseases of the vascular system; for it is only lately that, in estimating the forces which influence the circulation in diseased parts, they have begun to appreciate the preponderating influence of the capillary vessels, independently of the heart's action and the vis a tergo. It is only lately that they have recognised the important truth, that diseased vascular action may commence in circumference.

I am willing to allow that, in most cases of general paralysis, the affection of the muscular system is produced by disease of the nervous centres; yet I think it is also evident, that an injury of the extremities, or circumferential parts of the nerves, may cause such a derangement of their functions as to give rise to paralysis. The reason why persons seek for the explanation of paralytic symptoms by referring them to the nervous centres rather than their peripheral extremities is, because this mode of inference accounts more satisfactorily for the simultaneous affection of many parts of the system. Thus, if one hemisphere of the brain, or both, or if the cerebellum or spinal cord be pressed or injured, those parts which have a nervous connexion with them will experience a corresponding derangement of function. But if a process of disordered action be set up in one part of the nervous extremities, and this passes on to another part, the translation seems very strange, and you cannot easily comprehend why paralysis of one peripheral part will produce the same disease in another. It has been asked, whether a local paralysis ever can, by spreading towards the centre of the nervous system, produce paralysis in another and a distant locality.* This is a question we are not in the habit of investi-

* This question has not been discussed; although the French pathologists, when speaking of what they term the "paralysis of innervation," acknowledge the principle of paralysis existing independently of cerebral or spinal disease.

gating; and I think it has never been sufficiently or satisfactorily examined, considering its importance in a practical point of view, and the new light which it may throw on many of the most obscure and perplexing forms of disease. I shall endeavour to prove, first, that paralysis (from whatsoever cause it may arise) affecting one portion of the circumferential extremities of the nerves, may also affect other portions of their extremities; secondly, that pain originating in one situation may produce a similar sensation in distant parts; and thirdly, that convulsions resulting from irritation in any part of the extremities of the nervous system may occasion a corresponding train of symptoms in other parts of the body. You perceive, gentlemen, that I have enumerated the three most remarkable symptoms resulting from disease of the nervous system, namely, paralysis, pain, and convulsions. If I succeed in showing that each of these may be produced by causes acting on the extremities of the nervous system at a distance from the part affected, the position I have advanced will be proved.

A few days ago, happening to call at a gentleman's house, I was told by a young lady that she had wounded the inside of the ring-finger with a blunt needle, and that she found in it a considerable degree of numbness and loss of sensation. I said to her, "your little finger is also numb." You are aware those two fingers are supplied by the same branch of the ulnar nerve. Well, the little finger was really numb, as well as the finger next to it, which had been injured. What were the circumstances of the case in this instance? The side of the ring-finger next to the little finger had been wounded with a blunt needle; the impression made on the nervous extremities of the side of one finger produced numbness not only in that finger, but also the same cause operated backwards, or towards the centre, so as to affect the branch given off to supply the little finger, by the ulnar nerve, above the place of the wound. Here is an instance of a cause producing numbness of a particular branch of nerve, occasioning the same affection in another branch, and giving rise to phenomena identical with those which might arise from an injury of the main branch of the ulnar nerve. This is a plain fact. You have a case of precisely the same paralysis in a poor woman in this hospital, who has been complaining of rheumatic pains in various parts of her body. Before I had been struck by these and other instances of the same kind, I looked for the cause of this paralysis in the trunk; now I can understand how it may be in the periphery. You recollect I made some observations before on this subject, and mentioned that this numbness is frequently remarked in cases of gout and rheumatism, and that this occurrence in old persons often excites apprehensions of approaching paralysis. I have known old gentlemen so alarmed by it as to seek medical advice; and, as this affection sometimes precedes gout, and sometimes accompanies rheumatic arthritis and phlegmasia dolens, it is a fact worthy of your attention, and one which I would recommend you to hold in memory, though I must confess I am not able to give any explanation of it. I have seen an attack of this peripheral paralysis in a gentleman of gouty habit, and heard him express a great deal of surprise when he was told by Mr. Kirby, his medical attendant, that it would usher in a fit of his complaint. This gentleman, however, after taking some warm stimulant medicine, went to bed, and next morning had a regular attack of gout. But, to return to our subject. If you make experiments by handling snow, or immersing your hands in freezing mixtures, or any fluid of very low temperature, you find that, after some time, the exposed parts lose first the power of sensation, and afterwards that of motion, and that in this way you produce a complete, though temporary, local paralysis. Of this fact you are all aware. But what bears more strongly on the subject in question is that the paralysis thus induced is not merely confined to the hands and fingers, but also extends to other parts. You not only have the hands and fingers numb, but also lose, in a great degree, the power of flexion and extension, which is seated in the muscles of the fore-arm, and the motions of the wrist-joint are very imperfectly performed. Now all this time the muscles of the fore-arm, lying at a considerable depth, and covered by warm clothing, are protected from cold, and yet you perceive they partake in the paralytic affection of the exposed parts. Here, then, is another example of the same nature, corroborating our former position, that causes, producing loss of power in one part of the extremities of the nervous system, may have not merely a local influence, but also travel towards the centre and affect distant parts. Speaking of the influence of cold on the system, I have to observe, that, from the experiments made on this subject by Hunter, Edwards, Dr. Marshal Hall, and others, some instances of its effects seem very singular. One of the most remarkable is the production of paralysis, which, in most cases, is partial, but is sometimes very general without being followed by death. I remember the case of a dog, which lay buried in snow for two days, and was then taken out quite stiff and insensible, and thrown on a dunghill as if dead. After some time, the poor animal gave some symptoms of reanimation, and finally recovered. The influence of cold has been alluded to by Dr. Abercrombie; and you will find, that he mentions a case of paraplegia, arising from paralysis brought on by cold, which lasted for eight months. A blast of cold air on one side of the face has been known to cause paralysis and distortion of several months' duration. Again, you have, as in the case of a man in this hospital, paralysis of the lower extremities from exposing the feet to cold and wet, while employed in baling out water in a quarry. You may have observed the same thing brought on by similar exposure in fishing, or snipe-shooting, and that such causes gave rise to paralysis, not only in the parts subjected to the influence of diminished temperature and wet, but even extended to the nervous centres, so as to produce decided paraplegia. I was once myself exposed to a very intense degree of cold on board a ship, and observed that the sailors who had been most exposed suffered severely, and did not recover from its effects during the rest of our voyage. In fact, many months will often pass away before the symptoms arising from cold are removed, and you will find, that, in addition to the case of paraplegia from cold, which lasted eight months, Dr. Abercrombie mentions another, in which the paralysis was permanent.

One of the most remarkable examples of disease of the nervous system, commencing in the extremities, and having no connexion with lesions of the brain, or spinal marrow, was the curious *epidémie de Paris*, which occurred in the spring of 1828. Chomel has described this epidemic in the 9th Number of the Journal Hebdomadaire, and having witnessed it myself, in the months of July and August of the same year, I can bear testimony to the ability and accuracy of his description. It began (frequently in persons of good constitution) with sensations of pricking and severe pain in the integuments of the hands and feet, accompanied by so acute a degree of sensibility, that the patients could not bear those parts to be touched by the bed-clothes. After some time, a few days, or even a few hours, a diminution, or even abolition of sensation took place in the affected members, they became incapable of distinguishing the shape, texture, or temperature of bodies, the power of motion declined, and finally they were observed to become altogether paralytic. The injury was not confined to the hands and feet alone, but advancing with progressive pace, extended over the whole of both extremities. Persons lay in bed powerless and helpless, and continued in this state for weeks, and even months. Every remedy which the ingenuity of the French practitioners could suggest was tried, and proved ineffectual. In some, the stomach and bowels were deranged, and this affection terminated in a bad state of health, and even in death; in others, the vital organs, cerebral, respiratory, and digestive, were in the same state as before their illness, and their appetites were good, but still they remained paralytics. At last, at some period of the disease, motion and sensation gradually returned, and a recovery generally took place, although, in some instances, the paralysis was very capricious, vanishing and again re-appearing. The French pathologists, you may be sure, searched anxiously, in the nervous centres, for the cause of this strange disorder, but could find none; there was no evident lesion, functional or organic, discoverable in the brain, cerebellum, or spinal marrow. Now, here is another remarkable instance of paralysis creeping from the extremities towards the centre; here is a paralysis affecting all parts of the extremities as completely as if it had its origin in the central parts of the nervous system; and can any one, with such palpable evidences before him, hesitate to believe, that paralysis, or even hemiplegia, without any lesion of the brain, or spinal cord, may arise from disease commencing and originating in the nervous extremities alone? I may observe, *en passant*, that where paralysis simultaneously attacks the arm and leg of the same side, it arises from an impression on the nervous centres; but this, I think, does not hold where the paralysis is creeping, as in the case before me, which has been taken by Mr. Hudson, and is at present under the care of my colleague, Dr. Stokes. "The patient, James Moore, was admitted on the 3d of March, labouring under paraplegia, which he attributed to cold and wet. About a month before admission, he first perceived a stiffness of the great toe of the right foot, afterwards numbness and coldness of the sole, and then of the leg as far as the knee, and dragging of the limb in walking. During the progression of the disease up along the thigh, it commenced in the left foot, and, after a few days, he experienced almost complete paralysis of sensation in the right lower extremity, and a lesser degree in the left, accompanied by so much diminution of the power of motion, as to render him unable to walk without support. About three weeks after the appearance of paralysis in the lower extremities, the little finger of the right hand was attacked with numbness, which passed successively to the rest, attended by some loss of the sense of touch and power of grasping objects. He has also had retention of urine, and the bowels were obstinately constipated. There was no tenderness of any part of the spine. He had no pain in the head. His pupils were natural, mind unaffected, pulse, sleep, and appetite, also natural." Here, gentlemen, you have an instance of what I would term creeping paralysis, having its origin evidently in an affection of the peripheral extremities of the nerves.

I may now observe, that I have brought forward instances to prove, that direct injury of one part of the nervous system may produce paralysis in another and distant part; but have we not also other instances? Certain substances, which produce morbid effects on the nervous system, are found to be attended by results analogous to those described. You are all aware that lead frequently brings on paralysis; that this is caused by the local application of lead, and that the effect of the local application extends chiefly to those parts to which the lead is directly applied. Thus, in painter's cholic, the paralysis almost invariably begins in the hand and wrists, preceded, I will allow, in many cases, by symptoms of poisoning of the system, as shown by the tormina and affection of the intestinal canal. Dr. Bright has remarked, that, in painter's cholic, the spine is frequently tender in the cervical region, when the upper, and in the lumbar, when the lower, extremities are affected. It has been remarked, that spinal tenderness is often the consequence of disease of the extremities, and not the cause; so, I think, it is in painter's cholic. We found, in this hospital, a great number of cases, in which there was paralysis of the upper extremities without any spinal tenderness in the commencement; but when the disease had lasted for some time, the affection seemed to spread towards the spinal column. When this took place, it generally caused an aggravation of the disease; but it is no less true, that we had many instances where it could not be discovered; and you are not to think that this irritation of the spinal cord should always precede the paralytic affection of the wrist and hand, which is observed in painter's cholic. You have seen, in this hospital, two cases of spinal tenderness supervening on peritonitis and acute gastric irritation; and, in fact, in every disease in which the nervous extremities, which are distributed to the parietes, or viscera of the abdomen, you find almost invariably that, after some time, there will be pain and tenderness of the spinal column, as the consequence of those diseases. On the other hand, I grant, that as soon as the spine becomes affected, whether the disease be tympanitis, peritonitis, or that swelling of the belly to which the name of hysterical meteorism is applied, there will be certainly an aggravation of the existing symptoms.

You perceive this conducts us to the solution of the question, how far, in the treatment of chronic complaints, are we to consider spinal neuralgia as the cause or consequence of the disease. Sometimes those troublesome hysterical affections, which you are called on to treat, are preceded by spinal neuralgia, but in many well-marked cases it is totally absent. I wish to call your attention to this subject, because medical men have been biassed, to a very considerable extent, by the statements made by Mr. Teale, and others, respecting the treatment of various anomalous affections, supposed to be connected with irritation in the spinal column. Every female who complains of any kind of abdominal or pectoral symptoms of an obscure nature is examined all over the spine, and if the slightest tenderness be detected, according to the practice generally pursued, you are to leech and blister her back, or to apply tartar emetic ointment. I think I have seen injurious effects from this plan of treatment. Inquire carefully into the history of the case, and ascertain, if possible, whether it was the central or circumferential parts which were first affected, for, in the latter case, you can promise yourselves less from any local application to the spine than in the former; whereas, in those instances where the disease has travelled from the centre to the circumference, you may hope for success from local applications. It is important to recollect, gentlemen, that violent enteritic affections may produce paralysis of the lower extremities. In the case of a young gentleman, whose disease arose from obstruction in consequence of eating nuts, and to which I formerly adverted, violent enteritis and peritonitis arose, and he had two relapses; from these he recovered with difficulty, but they left him paralytic of his lower extremities. After two months, the paralysis speedily yielded to the application of stimulating liniments. This case Mr. Kirby and Mr. Cusack saw. In another remarkable case, concerning which I was consulted by Dr. Ireland, a frequently-recurring vomiting was in the end followed by a paralysis of the lower extremities; but as I mean to publish the particulars of this case, I shall say no more of it at present.

What I wish to impress upon your attention is, that pain, numbness, spasm, and loss of power, from an affection of the circumferential parts of the nerves, may commence in those extremities, and be propagated towards the centre, so as to be finally confounded with diseases originating in the central parts themselves. You have seen in the patient, James Moore, hemiplegia, which I am convinced had its origin in the extremities. Have you not also seen, in the cases of peritonitis, gastric irritation, and painter's cholic, a consecutive affection of the spine? Indeed, it frequently happens that paralysis, commencing in the nervous extremities, may not only induce disease of the spine, but in time bring on disease of the brain itself. It does not follow that a fatal paralysis affecting the brain should commence in that organ. In Dr. Woolaston's case, are we to account for the occasional partial amaurosis under which he laboured, for such a length of time before his death, by referring it to disease of the brain? In consequence of a temporary paralysis of one half of the retina of each side, he saw but the halves of objects, and from this he argued, that there was a semi-decussation of the optic nerves. This happened several times, but never remained for any length of time, and I do not think that at that period it was proved that any disease existed in the brain.* Some time back, I saw, with Dr. Brereton, a very singular example of defective vision in a wealthy bookseller, who had lost the sight of one eye from accident. This gentleman, one day, in going up a hill near Clonskeigh, remarked, that where there was but one man, he saw two men, but divided at the middle, as if they were cut by a vertical line into two halves. I questioned him closely on the occurrence, thinking it to be the effect of imagination, but he said this was not the cause, and that he was perfectly convinced he saw double. There is but one way of accounting for this optical delusion. It is well known that when vision is much impaired, the power of seeing light often remains, when the eye cannot distinguish any particular object. A partial and temporary paralysis of the retina, in a vertical section, may have given rise to an apparent white line, bisecting the object vertically. Again; in the case of a fine young lady, whom I saw along with Dr. Beatty, amaurosis, acute, sudden, and complete, came on without any headach or cerebral symptoms being complained of. When called to see her, I found her walking about the drawing-room, quite cheerful, and enjoying a good appetite, but perfectly blind. After the lapse of some days, these symptoms were followed by profound coma and death. But there are other instances more decidedly corroborative of the positions I have laid down. You all know that if a man gets a blow or cut on the forehead, which wounds or divides the frontal nerve, not only the parts which that nerve supplies become paralytic, but that also the diseased impression, thus produced, spreads towards the centre, affects those nerves which anastomose with the frontal, and, by means of the communication formed between the nerves of the eye-ball, through the lenticular ganglion, deranges the functions of the optic nerve and causes amaurosis. Formerly I was in the

* I do not lay much stress on this case, as the organic disease found after death was so considerable. In connexion with the next, however, it is interesting.

habit of giving a different account of this, and thought that, because, in some of the lower classes of animals, as for instance the mole, the fifth nerve, from which the frontal is derived, is the true nerve of vision, those animals having no optic nerve,* I had found an analogy capable of giving an explanation of the fact, that injury of the frontal nerve is sometimes followed by blindness. But this, I am of opinion, cannot be the true mode of accounting for the amaurosis, as I can now readily conceive how injury of any other nerve, having communication with the optic, may spread inwards, and finally derange or destroy its functions.

You will frequently observe persons in the decline of life, who otherwise enjoy tolerable health, exhibiting, as it were, a slight shade of paralytic affection of the system, fitful and capricious in its appearance and duration, sometimes remarked on every instance of corporeal exertion, sometimes scarcely at all, presenting at one time a reiteration of successive attacks, and at another time being totally absent for months. Some cases of this kind I have studied for months, and one in particular for years. The gentleman, who was the subject of the latter, complained of barely perceptible weakness, and dragging of one of his legs whenever he was tired; but, if he took a glass of wine on coming home, he got quite well, and these symptoms disappeared. Matters went on in this way for a considerable length of time, the paralysis being at one time in one leg and then in the other. At last he got a paralytic stroke, which lasted for some time, and then subsided. He next got confirmed paralysis of one side, and, soon after this, was carried off by an attack on the brain. You will often find persons similarly affected with paralytic attacks of the extremities; at first slight and transient, but afterwards increasing in vigour and intensity, until they terminate in ramollissement or effusion. Formerly I was of opinion, that this fugitive and shifting paralysis depended upon local congestion in the brain, and others have attributed it to effusion; but this is not the fact. Persons may die after having laboured for some time under hemiplegia, and yet no trace of lesion of the cerebral mass could be detected; and why? Because many of them are cases of this creeping paralysis, commencing in the peripheral extremities, and travelling gradually towards the centres of the nervous system.

It is only on the principle of there being such a disease as local paralysis not induced by lesions of the nervous centres, that we explain the origin and nature of such cases as paralysis of the deltoid, concerning which Dr. Elliotson has made so many interesting ob-

* A curious instance of the total absence, or imperfection, of a pair of nerves, is related by the Rev. Mr. Bree, in the Magazine of Natural History: "A white cat, of the Persian breed, was kept in his family as a favourite. The animal was a female, quite white, and perfectly deaf. She produced, at various times, many litters of kittens, of which some were quite white, others more or less mottled, tabby, &c. &c. But the extraordinary circumstance is, that of the offspring produced at one and the same birth, such as were like the mother, entirely white, were, like her, invariably deaf; while those that had the least speck of colour on their fur, as invariably possessed the usual faculty of hearing."

servations. It is by reference to this hypothesis alone, that we can account for the following cases, detailed by Dr. Cooke, in his admirable work on palsy:

"I have lately had an opportunity of seeing a case of anomalous hemiplegia, attended by circumstances not less extraordinary than those above described. An officer of high rank in the army, who is now about sixty years of age, was, in the year 1795, affected with a diminution of power in the right hand. This complaint increased, notwithstanding a variety of modes of treatment, till the year 1800; when, after a course of mercury, recommended by Mr. Cline, its further progress was stopped, since which time the disease has remained stationary. The peculiar circumstances of this case are the following. The muscles of the left arm, from the shoulder to the elbow, are much wasted, and greatly diminished in power; while the muscles of the fore-arm are not at all lessened in size, and but little in power. The state of the right side is just the reverse, the muscles of the upper arm being of their natural size, and possessing their full power; whilst those of the fore-arm are very much wasted, and their motion, especially that of the fingers, is almost entirely abolished. In all other respects, this gentleman appears to be perfectly well. No cause for this disease can be assigned, nor did any method of treatment afford the smallest relief, till the mercurial course was adopted, when the progress of the disorder was arrested in the year above-mentioned. Since that time no attempts to remove the complaint have been made, yet it does not increase.

"In a late publication by Mons. Keratry, a case of general palsy is related, the circumstances of which are very extraordinary. This case is adduced with a view of showing how little residue of animal existence is sufficient for the preservation of the intelligent being. There is now living, he says, in D'Isle et Vilaine, a person, who, after having been blind for ten years, lost also the sense of hearing, and, in a little time afterwards, became almost universally paralytic. He was entirely deprived of the use of his arms, legs, thighs, and of the whole exterior surface of the body, with the exception of a part of the face; but the power of speech, and the functions of respiration, circulation, and digestion, remained. Under these deplorable circumstances, however, he is not, says Mons. Keratry, wholly without consolation, for a sort of intercourse is preserved with his family and friends, by means of characters traced on that part which still retains its sensibility, and in this state of unexampled misery, he retains, in some degree, the distinguishing character of man—intelligence."*

I saw, the other day, with Mr. Crampton, a case of paralysis, in which the mouth was drawn upwards and to one side, accompanied by ptosis of the upper eyelid of the same side, so as to produce very great distortion. Mr. Crampton, with his usual decision, said,

* Inductions Morales et Physiologiques, p. 375.

"put a blister here and there, here and then there, and you set things to rights," marking out, at the same time, a space over each of the principal trunks of the fifth nerve, which are expanded over the side of the face. It happened exactly as he predicted; the first blister we applied pulled up the eyelid, the next partially rectified the distortion of the mouth, and the third made it quite straight. Now, the phenomena of this case and its treatment cannot be explained by supposing the paralysis to arise from disease of the brain; but if, on the other hand, you consider the disease as originating in the nervous extremities themselves, how easy will it be to account for the mode of operation.

So far of paralysis, gentlemen, for I perceive our time has expired. I trust, however, that I have brought forward a sufficient number of examples in proof of the position with which I set out, that diseases may commence in the circumferential parts of the nervous system, and may extend not only to other parts of the branches or extremities, but even travel towards the centre, and finally affect the brain or spinal cord. The consideration of this subject opens a vast field for investigation, and may, perhaps, form an additional clue to discovery of the nature and treatment of a most obscure and difficult class of diseases.

---

### PRACTICAL MEDICINE.

*Case in which a large Dose of Camphor was taken.* By G. Eickhorn, m.d. (Communicated in a Letter to Dr. Hays.)

I have read this morning, in the sixth volume of your Journal, page 236, a notice of a case in which a large dose of camphor had been taken; and as I have had an opportunity of experiencing in my own person the effects of such a dose of that article, and as the results in my case differed in many respects from the one you have noticed, a narrative of the symptoms I experienced may not be uninteresting.

In 1817, during the wet month of November, (in Germany,) I was attacked with cold in the head and a severe cough. One evening I took a lump of camphor, about the size of my thumb, and rubbed it down with sugar, with the intention of taking a little of it occasionally, and left it in the mortar covered with a sheet of paper; it was about six o'clock, I sat alone, and being unable to read, because my eyes were swimming in tears, time passed very tediously, and I therefore took without reflection, from time to time, a teaspoonful from under the cover without lifting it, till about nine o'clock, when I perceived the mortar almost empty; I had taken at least two-thirds of the whole. The idea then rushed to my mind that I had perhaps poisoned myself; but as I did not feel any ill effects, I resolved to wait till such symptoms should ensue as might call for interference. I accordingly went to bed, taking with me laudanum and diluted sulphuric acid. I had not been half an hour in bed before I began to feel warmer and warmer, till I

experienced a burning heat, and at the same time my heart throbbed more and more frequently till it was impossible to count the pulse, but unattended with any uneasiness in the head. I never felt better; never were my ideas more lively or clearer; it appeared as if my intellectual powers were increased, and certainly champagne never brought on a more pleasing intoxication. In this situation I passed about an hour and a half, or two hours, when my skin began to grow moist; soon after my pulse became slower, and I fell asleep. The next morning I awoke miserably weak, the sweat having penetrated to the lower side of the feather bed, and my shirt and clothes were drenched. My cold and cough were just as the evening before. I took a lump of camphor about the same size, and found it to weigh nearly ʒiij., and supposing the powder left in the mortar to have been the third part, I had taken ʒij. or 120 grains.

The difference between the two cases as to the symptoms is indeed great; in the case at Breslau the pulse was hard; in mine this did not occur, and the frequency was so great, that I cannot conceive how the pulse could have become hard; then, in the case alluded to, the pulse was full; in mine it was small in accordance with its frequency; there was heaviness of the head; my head felt rather light: anxiety and agitation were experienced in the other instance; I have never felt more exhilarated and comfortable: the individual at Breslau suffered violent heat in the stomach; I did not experience any uneasy sensation in that organ; and, as to the senses, I remember only that I could perceive the pulsation of the arteries in the ear, but without any disagreeable sensation. I do not recollect any derangement in the function of the bladder, and therefore suppose there was none. The difference in the symptoms may be accounted for by my having taken the camphor unmixed with any article capable of modifying its effects, whilst by the individual at Breslau the substance was taken dissolved in four ounces of brandy, to which last article I think many of the symptoms are to be ascribed.—*American Journal of the Med. Sciences.*

---

### PRACTICAL SURGERY.

### *External Iliac Artery successfully tied.* By JAMES C. HALL, M.D., of Washington City.

THE feasibility of tying the external iliac artery is so well established, that the following operation is deemed worthy of record only from its having been performed under circumstances extremely unfavorable to success.

Oct. 5, 1831. I saw George Snow, a labourer, age thirty-five, of intemperate habits; now confined to his bed; reduced in flesh; haggard in appearance; digestive functions impaired. He relates, that three months since, in lifting a heavy burden, he felt something give way in his thigh, and in two or three days afterwards he noticed a small tumor in the upper and inner part of the limb, and that it has unremittingly continued to increase. At this time the left thigh is four times by admeasurement the size of the other, and the leg, ankle, and foot are highly œdematous and varicose. Upon the anterior part of the thigh is presented a tumor, extending from Poupart's ligament, and the superior spine of the ilium, one third or more of the way downwards, elastic, resisting and strongly pulsatile; it is of a dark-bluish colour, and this not truly pointed; has a prominence when the impulse of the blood is forcibly felt, and produces the impression that here the parietes are very thin. The pulsation is evident on a very wide surface, and by means of the stethoscope is discovered, high up in the iliac fossa, where also there is much fulness. Here two symptoms give the case a formidable and almost desperate character, rendering it very doubtful whether the rupture in the artery has occurred above or below Poupart's ligament; but, as the pressure of the finger upon the iliac artery arrests the pulsation, and as the prominence of the tumor is below the groin, I feel authorized in thinking that the disease is diffused aneurism arising from a rupture of the femoral artery; that the iliac artery is sound, and may therefore be safely tied. This day the temperature of the affected limb is 84° between the toes, and 86° at the inner part of the thigh, that of the sound limb is 94° and 96° at the corresponding points. There is a general numbness of the thigh, with a severe and constant pain about the knee; pulse ninety.

Oct. 6th. Pulse 114; in other respects no change.

Oct. 7th. Drs. Dacoes, Warfield, and Collins, examined the case, and concurred in opinion with me that the operation of tying the external iliac artery gave the only hope of escape from an early death.

This operation I proceeded immediately to perform in the mode recommended by Sir A. Cooper; but, as the processes of the ilium and the line of Poupart's ligament were completely obscured by the swelling, the location of my incision was necessarily uncertain. It commenced at a point opposite to and distant from the superior spine of the ilium about one inch, and extending with a gentle curve downwards, terminated on the outer side of the external abdominal ring. The artery was found, and proved to be sound, but great difficulty was experienced in passing the ligature, owing to the very great toughness of the substance connecting it with the vein. The needle in Physick's artery-forceps twisted and broke in attempting to pierce this fascia, and finally I was compelled to pass a knife-handle under the artery, upon the extremity of which I cautiously made an incision. With Gibson's instrument I then passed the ligature, which was tied singly. Four other vessels were tied. The peritoneum was not injured.* The pulsation in the tumor

* The great strength of the living peritoneum is proved by the following singular accident. Mr. Rodney, age twenty-five, sitting on a boy's sled, rapidly descended a frozen surface, and came in contact with a blunt stick, an inch immediately ceased, but there was no perceptible diminution in size. The wound was dressed simply; the whole limb invested in carded cotton, and laid in a flexed position.

Three hours after operation, pulse 120; temperature of left limb 85°; of the other 91°; has some griping near the wound; limb numb; pain at knee severe, insensible to friction, general aspect natural. At one o'clock A.M. slight bleeding at nose; shortly after slept.

Oct. 8th. At seven A.M. complained of a "heaving" sensation, which was relieved by a mouthful of weak toddy. At eleven A.M. feels well; aspect natural; no appetite; no thirst; tongue clear; pulse 116. Pain in knee diminished, but yet severe, circumference on tumor the same; other parts of limb much diminished; veins moderately full, temperature in both limbs 87°. At two P.M. great agony in limb, which is affected in succession with the sensations of hot embers being upon it, of the pouring of hot water, and the pricking of many needles; complains of nausea and giddiness.

Oct. 9th. Limb much reduced, but not the tumor; numbness ceased; pain much relieved; pulsation thought to be discovered in the anterior tibial artery. Wound nearly closed through its whole extent, having only a little pus and flakes of lymph upon its edges.

At six P.M., after the operation of oil taken in the morning, he complained of a sensation in the abdomen "like that of a 50lb. weight going up and down," and, upon the hand being applied, the aorta could be felt strongly beating. At ten P.M. laudanum was given to arrest the operation of the oil and calm his irritation.

Oct. 10th. Pulse varied from 100 to 116; temperature nearly equal in both limbs. Applied frictions and warm envelopes to remove coldness of leg.

Oct. 11th. Beating sensation above wound.

Oct. 12th. Not so well; wound sore; "much beating and throbbing in middle of leg."

Oct. 15th. His bowels disordered; dark and fœtid stools. Ordered calomel and opium.

From this time no change worthy of record occurred. The œdema rapidly disappeared, while the tumor formed by the coagulated blood very slowly decreased under the use of stimulating frictions. The pain of the knee, depending probably upon pressure made upon the nerves, could only be alleviated. The wound remained generally in a healthy condition, though the healing process was not as rapid as at first promised. Upon the 22d day the ligature came away. The patient continued to do well in every respect, and, though the limb is not entirely reduced to its natural broad and half an inch thick: it entered by the side of the rectum, and penetrated six inches. He withdrew it himself: there was a gush of blood; he fainted, and died in thirty-one hours. I did not see him till after death, when, upon examination, I found that the stick had passed between the rectum, peritoneum, and sacrum, and had wounded the primitive iliac just at its bifurcation. The peritoneum was not wounded at all.

standard, no doubt remains that it will be perfectly restored. To the assiduity and skill of Dr. Barry, who attended during the after-treatment, I am much indebted for the happy termination of this case.—*Ibid.*

### INTELLIGENCE.

### THE EPIDEMIC INFLUENZA.

